# Particulate Matter Exposures, Mortality, and Cardiovascular Disease in the Health Professionals Follow-up Study

**DOI:** 10.1289/ehp.1002921

**Published:** 2011-03-31

**Authors:** Robin C. Puett, Jaime E. Hart, Helen Suh, Murray Mittleman, Francine Laden

**Affiliations:** 1 South Carolina Cancer Prevention and Control Program and Department of Environmental Health Sciences, Arnold School of Public Health, University of South Carolina, Columbia, South Carolina, USA; 2 Exposure, Epidemiology, and Risk Program, Department of Environmental Health, Harvard School of Public Health, Boston, Massachusetts, USA; 3 Channing Laboratory, Department of Medicine, Brigham and Women’s Hospital and Harvard Medical School, Boston, Massachusetts, USA; 4 Environmental Health Program, National Opinion Research Center at the University of Chicago, Boston, Massachusetts, USA; 5 Department of Epidemiology, Harvard School of Public Health, Boston, Massachusetts, USA; 6 Cardiovascular Epidemiology Research Unit, Beth Israel Deaconess Medical Center, Harvard Medical School, Boston, Massachusetts, USA

**Keywords:** air pollution, cardiovascular disease, mortality, particulate matter

## Abstract

Background: The association of all-cause mortality and cardiovascular outcomes with air pollution exposures has been well established in the literature. The number of studies examining chronic exposures in cohorts is growing, with more recent studies conducted among women finding risk estimates of greater magnitude. Questions remain regarding sex differences in the relationship of chronic particulate matter (PM) exposures with mortality and cardiovascular outcomes.

Objectives: In this study we explored these associations in the all-male Health Professionals Follow-Up Study prospective cohort.

Methods: The same spatiotemporal exposure estimation models, similar outcomes, and biennially updated covariates were used as those previously applied in the female Nurses’ Health Study cohort.

Results: Among 17,545 men residing in the northeastern and midwestern United States, there were 2,813 deaths, including 746 cases of fatal coronary heart disease (CHD). An interquartile range change (4 µg/m^3^) in average exposure to PM ≤ 2.5 µm in diameter in the 12 previous months was not associated with all-cause mortality [hazard ratio (HR) = 0.94; 95% confidence interval (CI), 0.87–1.00] or fatal CHD (HR = 0.99; 95% CI, 0.87–1.13) in fully adjusted models. Findings were similar for separate models of exposure to PM ≤ 10 µm in diameter and PM between 2.5 and 10 µm in diameter and for copollutant models.

Conclusions: Among this cohort of men with high socioeconomic status living in the midwestern and northeastern United States, the results did not support an association of chronic PM exposures with all-cause mortality and cardiovascular outcomes in models with time-varying covariates. Whether these findings suggest sex differences in susceptibility or the protective impact of healthier lifestyles and higher socioeconomic status requires additional investigation.

The association of ambient particulate matter (PM) exposures with mortality and cardiovascular outcomes has been well established in studies of short-term exposure (Committee on the Medical Effects of Air Pollutants 2006; Levy et al. 2000; Samet et al. 2000a, 2000b; Zanobetti et al. 2000). The literature on health outcomes associated with long-term PM exposures has been growing. Beginning with the Harvard Six Cities Study and the American Cancer Society Study, researchers have reported increased risks of all-cause and cardiopulmonary mortality with chronic exposures (Dockery et al. 1993; Pope et al. 1995). More recent cohort studies have confirmed these findings (Brunekreef et al. 2009; Eftim et al. 2008; Laden et al. 2006; Miller et al. 2007; Pope et al. 2004; Puett et al. 2008, 2009).

A few recently published cohort studies have focused on women and have reported stronger effect estimates for fatal coronary heart disease (CHD) and all-cause mortality than previous studies (Miller et al. 2007; Puett et al. 2008, 2009).

Questions remain regarding sex differences in the associations of chronic PM exposures with all-cause and cardiovascular mortality. In the present study we applied the same spatiotemporal exposure modeling used in the all-female Nurses’ Health Study cohort to examine similar relationships among the all-male Health Professionals Follow-Up Study cohort.

## Materials and Methods

*Study population.* The Health Professionals Follow-Up Study cohort consists of 51,529 male dentists, pharmacists, optometrists, podiatrists, osteopaths, and veterinarians residing in the United States. The cohort began with a baseline questionnaire in 1986, when participants were 40–75 years of age. The Health Professionals Follow-Up Study was approved by the Harvard School of Public Health Institutional Review Board, and returning the completed questionnaires constituted implied consent to use the data in ongoing research. Follow-up questionnaires are mailed every 2 years, with response rates of approximately 93%. The current study is limited to participants living in 13 contiguous northeastern and midwestern states (Maine, Vermont, New Hampshire, Massachusetts, Rhode Island, Connecticut, New York, New Jersey, Delaware, Pennsylvania, Ohio, Michigan, and Maryland) for comparison with our recent studies in the Nurses’ Health Study. Health professionals were excluded for any time period during which they lived outside this region.

*Outcomes.* The outcomes for the current study were all-cause mortality (excluding accidental deaths), nonfatal myocardial infarction (MI), fatal CHD, and hemorrhagic and ischemic stroke. We also examined total cardiovascular disease (CVD) events including fatal CHD, nonfatal MI, and total strokes. Deaths were confirmed by families, postal officials, and the National Death Index. We included MIs if medical record information met World Health Organization criteria (presence of symptoms and typical electrocardiographic changes or elevated cardiac enzyme levels) or if the patient was hospitalized and additional corroborating correspondence was obtained confirming the diagnosis (Rose and Blackburn 1982). Men with MIs before baseline were excluded. Fatal CHD was confirmed through hospital records or by a death certificate listing CHD as the primary cause or the most plausible cause and previous history of the disease were available. Strokes were included if medical records showed evidence of typical neurologic deficit of sudden or rapid onset lasting ≥ 24 hr attributed to a cerebrovascular event or if the participant was hospitalized and the supplementary correspondence corroborated the diagnosis. Hemorrhagic and ischemic strokes were categorized using National Survey of Stroke criteria (Walker et al. 1981). Men who experienced strokes before baseline were excluded. All medical records were reviewed by physicians blinded to exposure status of the participants.

*Exposure assessment.* Spatiotemporal models were developed to estimate monthly exposures to PM ≤ 2.5 µm in diameter (PM_2.5_) and PM ≤ 10 µm in diameter (PM_10_) for each address of each participant. Exposures to PM between 2.5 and 10 µm in diameter (PM_10–2.5_) were estimated for each month and location by subtracting the PM_2.5_ modeled estimates from the PM_10_ modeled estimates. These models allowed us to predict chronic PM exposure levels for all individuals with a geocoded address, even those living in areas with no nearby monitors. The models are described in detail elsewhere (Paciorek et al. 2009a; Yanosky et al. 2008, 2009). Briefly, the models use a geographic information system (GIS)-based spatial smoothing model and include pollution data from monitoring sites in the U.S. Environmental Protection Agency Air Quality System (AQS) (U.S. Environmental Protection Agency 2009), the Visibility Information Exchange Web System (VIEWS 2004), the Interagency Monitoring of Protected Visual Environments (IMPROVE) network, Stacked Filter Unit (a predecessor to IMPROVE), Clean Air Status and Trends (CASTNet) networks (Paciorek et al. 2009a, 2009b; Yanosky et al. 2008, 2009), and Harvard research studies (Spengler et al. 1996; Suh et al. 1997), as well as GIS-derived covariates including population density, distance to nearest road, elevation, urban land use, point-source and area-source primary emissions, and meteorological information. To allow for sparse PM_2.5_ monitoring before 1999, in previous studies separate generalized additive mixed models were constructed for the time periods from 1988 to 1998 and from 1999 to 2002 (Paciorek et al. 2009a; Yanosky et al. 2008, 2009). To compensate for the relative lack of PM_2.5_ data, the pre-1999 model added extinction coefficients and a simpler spatiotemporal structure to estimate the PM_2.5_ to PM_10_ ratio seasonally. Point-source PM_2.5_ emissions were included in the post-1999 PM_2.5_ model. The models were evaluated using cross-validation; they exhibited little bias and high precision (Paciorek et al. 2009a; Yanosky et al. 2008, 2009).

*Evaluation of confounders and effect modifiers.* Questionnaires were originally mailed in January 1986, and follow-up questionnaires were sent in January of subsequent even-numbered years. Data from these biennial questionnaires were used to assess potential confounding and effect modification by covariates. We selected covariates *a priori* based on known risk factors for mortality and CVD in the general population and specific to this cohort (Chiuve et al. 2008; Cho et al. 2002; Pischon et al. 2005). We examined confounding by adjustment and effect modification through the use of interaction terms and stratification for hypertension (yes, no), hypercholesterolemia (yes, no), diabetes mellitus (yes, no), physical activity [< 3; 3 to < 9; 9 to < 18; 18 to < 27; or ≥ 27 metabolic equivalents (MET)hr/week], alcohol consumption (0; 0.1–4.9; 5.0–14.9; ≥ 15 g/day), body mass index (BMI, continuous), smoking status (never, former, or current), smoking pack-years, and low- versus high-risk diet. A low-risk diet was assessed as a diet with a high ratio of polyunsaturated to saturated fat, high in cereal fiber, and low in trans fat and glycemic load (details described elsewhere) (Hu et al. 2001). Family history of MI was examined on the baseline questionnaire. On the baseline questionnaire, information was also available to determine if the mailing address provided was a residential or business location.

*Statistical analysis.* We used time-varying Cox proportional hazards models to assess the relationships of all-cause mortality and cardiovascular outcomes with predicted PM_10_, PM_2.5_, and PM_10–2.5_ exposures averaged over the 12 months before each outcome. All survival models were based on a monthly time scale and were used to estimate hazard ratios (HRs) and 95% confidence intervals (CIs). Person-years of follow-up time were calculated from baseline (January 1989) until the end of follow-up (January 2003). Person-time spent living outside the geographic region of interest was excluded, as were men with cardiovascular outcomes before baseline. Incidence rates were estimated as the number of new cases divided by person-years of follow-up. Each particulate size fraction was assessed in separate and combined models. Cox models were stratified by age in months to control finely for age in a nonparametric way. Models were also adjusted for year (linear term), season (indicator variables), and for state of residence (indicator variables), rather than stratifying by these covariates, to maximize the number of cases in strata. Effect modification was assessed through the use of interaction terms in the models, as well as through likelihood ratio tests (LRT). In a sensitivity analysis, we compared model results for each size fraction of PM for additional time windows of exposure: 24, 36, and 48 months. We also conducted sensitivity analyses excluding participants who resided outside metropolitan statistical areas (MSAs). All statistical analyses used SAS version 9.2 (SAS Institute Inc., Cary, NC).

## Results

**Figure 1:**
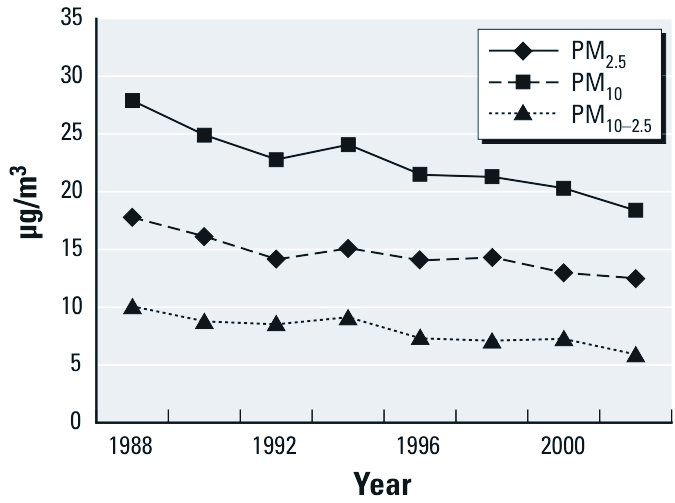
Mean annual estimated PM exposures from 1988 to 2002 in the northeastern and midwestern United States.

A total of 17,545 men were included at baseline, with a mean age of approximately 57 years (Table 1). Most were never or former smokers at baseline, with current smokers decreasing to about 5% of the study population by the end of follow-up. The percentages of men who reported hypertension, hypercholesterolemia, or diabetes increased considerably from baseline (26.5, 24.3, and 4.3%, respectively) to the end of follow-up (47.5, 56.6, and 9.8%, respectively). About 33% of the men reported ≥ 27 MET hr/week of physical activity in 1989, with the percent of respondents in this category growing by the end of follow-up. About 23% of the study population lived in New York, with 15% in Pennsylvania, 14% in Michigan, 13% in Ohio, and 10% in New Jersey. At the beginning of the study, the average annual estimated exposure was 27.9 µg/m^3^ for PM_10_, 17.8 µg/m^3^ for PM_2.5_, and 10.1 µg/m^3^ for PM_10–2.5_. In general, annual predicted PM exposures decreased over the follow-up period, with PM_10_ showing the largest decline (Figure 1). We performed correlations among various time windows of average exposure (12, 24, 36, and 48 months) for each PM fraction. All time windows were highly correlated (ρ > 0.86).

**Table 1:** Characteristics for the Health Professionals Follow-Up Study at baseline for the current study in 1989.

Variable	Value
*n*	17,545
Age (years)	57.4 ± 9.8
BMI	25.8 ± 3.4
Smoking status (%)	
Never	44.9
Current	9.5
Former	45.5
No. of pack-years	25.2 ± 19.4
Hypertension (%)	26.5
Hypercholesterolemia (%)	24.3
Diabetes (%)	4.3
Family history of MI (%)	14.0
Low-risk diet (%)	42.5
Alcohol consumption [g/day (%)]	
0	20.8
0.1–4.9	27.5
5.0–14.9	28.1
≥ 15.0	23.6
Physical activity [hr/week (%)]	
< 3 MET	14.9
3 to < 9	19.1
9 to < 18	19.0
18 to < 27	13.8
≥ 27	33.2
Predicted PM_10_ (µg/m^3^)	
Mean ± SD	27.9 ± 5.8
Interquartile range	7.4
Predicted PM_2.5_ (µg/m^3^)	
Mean ± SD	17.8 ± 3.4
Interquartile range	4.3
Predicted PM_10–2.5_ (µg/m^3^)	
Mean ± SD	10.1 ± 3.3
Interquartile range	4.3
Values are mean ± SD or percent unless otherwise noted. Percentages are based on complete data for participants.	

There were 2,813 deaths, 746 cases of fatal CHD, 646 cases of nonfatal MI, 1,661 cases of total CVD, 230 ischemic strokes, and 70 hemorrhagic strokes (Table 2). An interquartile range (4 µg/m^3^) change in average PM_2.5_ exposure in the 12 previous months was not associated with all-cause mortality (HR = 0.96; 95% CI, 0.90–1.03) or ischemic strokes (HR = 0.84; 95% CI, 0.67–1.06) in basic models adjusting for time and state of residence and stratified by age. The HR for fatal CHD was 1.01 (95% CI, 0.89–1.15), 1.02 for total CVD (95% CI, 0.94–1.11), and 1.06 for nonfatal MI (95% CI, 0.92–1.22) in similar models. For comparison purposes with other studies, translating these relative risks (RRs) to a 10-µg/m^3^ unit change in PM_2.5_ exposure results in an HR for all-cause mortality of 0.90 (95% CI, 0.76–1.06), HR for fatal CHD of 1.02 (95% CI, 0.74–1.41), and HR for total CVD of 1.05 (95% CI, 0.85–1.30). The HR for an interquartile range change in average PM_2.5_ exposure hemorrhagic strokes was highest, but the number of cases was small (HR = 1.14; 95% CI, 0.79–1.65). The HRs were very similar in fully adjusted models with additional adjustment for BMI, hypertension, hypercholesterolemia, diabetes, family history of MI, smoking (status and pack-years), physical activity, healthy diet, and alcohol consumption. For example, the fully adjusted HR for total CVD was 1.01 (95% CI, 0.93–1.10). Results were similar for interquartile range increases in PM_10_ (7 µg/m^3^) and PM_10–2.5_ (4 µg/m^3^) separately, as well as in copollutant models with PM_2.5_ and PM_10–2.5_ (Table 3).

**Table 2:** HRs (95% CIs) for associations of an interquartile-range change in average predicted PM exposure, adjusting for covariates.

PM_2.5_ (IQR = 4 µg/m^3^)			PM_10_ (IQR = 7 µg/m^3^)			PM_10–2.5_ (IQR = 4 µg/m^3^)		
Outcome	Cases	Person-years	Basic model*a*	Full model*b*	Basic model*a*	Full model*b*	Basic model*a*	Full model*b*
All-cause mortality		2,813		220,552		0.96 (0.90–1.03)		0.94 (0.87–1.00)		0.97 (0.91–1.03)		0.94 (0.89–1.00)		0.98 (0.93–1.04)		0.96 (0.91–1.02)
Fatal CHD		746		220,562		1.01 (0.89–1.15)		0.99 (0.87–1.13)		1.04 (0.93–1.16)		1.01 (0.90–1.14)		1.05 (0.94–1.16)		1.02 (0.92–1.14)
Nonfatal MI		646		214,919		1.06 (0.92–1.22)		1.06 (0.92–1.22)		1.05 (0.92–1.18)		1.04 (0.92–1.18)		1.03 (0.92–1.15)		1.02 (0.91–1.15)
Total CVD		1,661		212,649		1.02 (0.94–1.11)		1.01 (0.93–1.10)		1.04 (0.96–1.12)		1.02 (0.95–1.10)		1.04 (0.97–1.11)		1.03 (0.96–1.11)
Ischemic strokes		230		218,944		0.84 (0.67–1.06)		0.86 (0.68–1.08)		0.97 (0.79–1.19)		0.98 (0.80–1.21)		1.08 (0.90–1.29)		1.08 (0.90–1.30)
Hemorrhagic strokes		70		220,292		1.14 (0.79–1.65)		1.17 (0.81–1.69)		0.99 (0.70–1.41)		1.01 (0.71–1.44)		0.88 (0.63–1.24)		0.90 (0.64–1.26)
**a**Stratified by age in months, adjusting for year, season, and state of residence. **b**Stratified by age in months, adjusting for year, season, state of residence, BMI, hypertension, hypercholesterolemia, diabetes, family history of MI, smoking (status and pack-years), physical activity, healthy diet, and alcohol consumption.																

**Table 3:** HRs (95% CIs) for associations of interquartile-range (4 µg/m^3^) changes in average predicted copollutant (PM_2.5_ and PM_10–2.5_ ) exposures, adjusting for covariates.

All-cause mortality (2,813 cases, 220,552 person-years)	Fatal CHD (746 cases, 220,562 person-years)	Nonfatal MI (646 cases, 214,919 person-years)	Total CVD (1,661 cases, 212,649 person-years)	Ischemic strokes (230 cases, 218,944 person-years)	Hemorrhagic strokes (70 cases, 220,292 person-years)
Basic model*a*												
PM_2.5_		0.96 (0.89–1.04)		0.97 (0.83–1.13)		1.06 (0.90–1.25)		0.99 (0.90–1.10)		0.74 (0.56–0.96)		1.33 (0.86–2.06)
PM_10–2.5_		1.00 (0.94–1.06)		1.06 (0.94–1.20)		1.00 (0.88–1.14)		1.04 (0.96–1.13)		1.24 (0.99–1.54)		0.77 (0.52–1.15)
Full model*b*												
PM_2.5_		0.94 (0.87–1.02)		0.97 (0.83–1.13)		1.06 (0.90–1.25)		0.99 (0.90–1.10)		0.76 (0.58–0.99)		1.35 (0.87–2.09)
PM_10–2.5_		0.98 (0.92–1.05)		1.04 (0.92–1.17)		1.00 (0.87–1.14)		1.03 (0.95–1.12)		1.22 (0.98–1.52)		0.78 (0.53–1.16)
**a**Stratified by age in months, adjusting for year, season, and state of residence. **b**Stratified by age in months, adjusting for year, season, state of residence, BMI, hypertension, hypercholesterolemia, diabetes, family history of MI, smoking (status and pack-years), physical activity, healthy diet, and alcohol consumption.												

Effect modification was evident for PM_2.5_ associations (Table 4). Men without a family history of MI were at significantly lower risk for all-cause mortality associated with an interquartile range increase in chronic fine particulate exposure compared with men with such a history. Analysis of PM_2.5_ exposures and fatal CHD stratified by smoking status show an increased risk for fatal CHD associated with chronic fine particulate exposure among never smokers. Although the HR for current smokers was higher than that estimated for never smokers, there was only a small number of current smokers with fatal CHD, and there was no apparent relationship for former smokers.

**Table 4:** HRs for all-cause mortality and fatal CHD associated with an interquartile-range (4 µg/m^3^) change in average 12-month PM_2.5_ exposure, stratified by MI family history and smoking status, respectively.*a*^,b^

Variable	Cases	Person-years	HR (95% CI)	LRT *p*-value
Family history of MI		All-cause mortality						
No		2,439		189,619		0.91 (0.85–0.98)		< 0.01
Yes		374		30,933		1.08 (0.91–1.29)		
Smoking status		Fatal CHD						
Never		195		86,571		1.28 (1.01–1.63)		0.04
Former		398		95,384		0.90 (0.77–1.06)		
Current		47		13,550		1.40 (0.88–2.21)		
**a**Modeled stratifying by age in months, adjusting for state of residence, year, and season, smoking status, hypercholesterolemia, diabetes, hypertension, BMI, diet, alcohol consumption, and physical activity. **b**Results presented for complete smoking data.								

Sensitivity analyses showed that model results were stable using different time windows of average exposure for each size fraction of PM, as well as excluding state of residence from the final models. Findings also did not change when participants residing outside of MSAs were excluded or when the addresses used for exposure assessment were limited to those we were able to confirm as residential (data not shown). Finally, we conducted sensitivity analyses excluding men with prior cancers (other than nonmelanoma skin cancer) as well as including men with prior stroke and CHD, and these results were also comparable (data not shown).

## Discussion

Among men participating in the Health Professionals Follow-up Study cohort living in the northeastern and midwestern United States, we found no evidence of an association between all-cause mortality and an interquartile-range increase in estimated exposure to PM_2.5_, PM_10–2.5_, or PM_10_ averaged over the prior 12 months. We observed similar results for total CVD and fatal CHD with all size fractions of particulate exposures and with PM_2.5_ and PM_10_ for ischemic strokes. Risk of ischemic stroke and risk of nonfatal MI were slightly elevated with increases in PM_10–2.5_ and with all sizes of particulate exposures, respectively.

In contrast to other cohort studies of chronic exposures among men and women combined, the present study showed essentially no association. Both the original and extended follow-up of the Harvard Six Cities Study found all-cause and cardiopulmonary mortality significantly associated with long-term exposures to PM_2.5_, with increased risks for all-cause mortality ranging from 13 to 16% and cardiopulmonary risks from 18 to 28% for each 10-µg/m^3^ increase in PM_2.5_ (Dockery et al. 1993; Laden et al. 2006). The original and extended American Cancer Society Study included data collected as part of the nationwide Cancer Prevention Study II, restricting the approximately 1.2 million participants to those living in metropolitan areas with available pollution data. Reported risks associated with PM_2.5_ were lower than those from the Harvard Six Cities Study but still statistically significant (ranging from 4 to 6% for all-cause and from 6 to 9% for cardiopulmonary mortality per 10-µg/m^3^ increase in PM_2.5_) (Pope et al. 1995, 2002). A recent reanalysis of the American Cancer Society Study exploring adjustment for ecologic covariates also found an increase when adjusting for median household income (per 10-µg/m^3^ increase in PM_2.5_ RR for all-cause mortality: 1.05; 95% CI, 1.03–1.07) compared with no adjustment for ecologic covariates (RR for PM_2.5_: 1.03; 95% CI, 1.02–1.05) (Krewski et al. 2009). In the Medicare Cohort Air Pollution Study, Zeger et al. (2008) examined the relationship of all-cause mortality with a 6-year average exposure to fine particulates in ZIP code regions of the eastern region of the United States, which encompassed the geographic area of our study. The risk associated with a 10-µg/m^3^ increase in PM_2.5_ exposure was 6.8% (95% CI, 4.9–8.7%), adjusting for socioeconomic status (education, poverty, unemployment, income) and chronic obstructive pulmonary disease. Results were stronger for the central United States (13.2; 95% CI, 9.5–16.9) and lower for the western United States (–1.1; 95% CI, –3.0 to 0.8). Results from the Netherlands Cohort Study (NCLS) of men and women were similar to American Cancer Society Study findings (Brunekreef et al. 2009). The risk of CVD mortality was 1.04 (95% CI, 0.90–1.21) with a 10-µg/m^3^ increase in PM_2.5._ However, the same risk for an embedded case-cohort study was 0.83 (95% CI, 0.60–1.15), adjusting for a much larger number of potential confounders. Finally, similar to our findings, a study of male veterans (Lipfert et al. 2006) did not find evidence of an association between PM_2.5_ (mean ± SD, 14.3 ± 3.0 µg/m^3^) and all-cause mortality; however, the study found an association with traffic density, suggesting that traffic-related pollution may be more important in men.

Differences between our study findings and previously published results may stem from differences in the populations examined, pollution measures, and covariates considered in analyses. For example, city-specific PM_2.5_ averages in the Harvard Six Cities Study ranged from 11.4 to 29.0 µg/m^3^ in the original time period (Dockery et al. 1993) and from 10.2 to 22.0 µg/m^3^ in the extended period (Dockery et al. 1993; Laden et al. 2006), which includes our range of average estimated exposure from baseline (17.8 µg/m^3^) to the final year of follow-up (12.5 µg/m^3^). However, the Harvard Six Cities Study population exhibited higher percentages of smokers, a lower level of educational attainment, and between 28 and 53% occupationally exposed to dust/fumes, factors that may increase susceptibility to ambient air pollution exposures. Metropolitan area PM_2.5_ measures were used to estimate exposure in the American Cancer Society Study (Pope et al. 2002), which reported an average participant exposure of 17.7 µg/m^3^, similar to our study average. As with the Harvard Six Cities Study (Dockery et al. 1993; Laden et al. 2006) and the American Cancer Society Study (Pope et al. 2002), pollution levels did not differ greatly between our study and the Medicare Cohort Air Pollution Study (Zeger et al. 2008). Using a 6-year average exposure estimated at the ZIP code level, the interquartile range of PM_2.5_ exposure (12.3–15.3 µg/m^3^ for Eastern region) was lower than our baseline interquartile range (15.6–19.9 µg/m^3^). Reported RRs in this study were higher than those in our study, with models adjusting for fewer and area level covariates: socioeconomic status and chronic obstructive pulmonary disease. The Medicare Cohort Study population was also of lower socioeconomic status than the health professionals; however, Zeger et al. (2008) reported that analyses stratified by the national median of socioeconomic status were similar. Results from the NCLS (Brunekreef et al. 2009) were more comparable with our findings, and similar to our study, they adjusted for a much larger number of potential confounders than previous studies. Full cohort analyses were adjusted for age, sex, smoking, area level income, and area. Case-cohort analyses, which showed lower RRs, were adjusted for age, sex, weight/height squared, smoking, marital status, diet, alcohol use, education, occupation, and area-level income. In addition, pollution exposure methods included the use of updated residential addresses. We also note that although we have presented major findings in interquartile range change as well as 10-µg/m^3^ increments, some direct comparisons between previously published study findings and ours are limited because of differences in the units used for incremental change.

Few studies of chronic air pollution exposures have examined cerebrovascular outcomes. In the American Cancer Society Study, Pope and colleagues reported an increased risk of 2% (95% CI, –5 to 10%) for cerebrovascular death associated with a 10-µg/m^3^ increase in fine particulate exposures, which falls between our risk estimates for incident ischemic and hemorrhagic strokes with an interquartile-range change in average PM_2.5_ (4 µg/m^3^) (Pope et al. 2004). Among a national cohort of women (the Women’s Health Initiative), findings for first stroke associated with PM_2.5_ exposures were stronger, with a reported HR of 1.28 (95% CI, 1.02–1.61) for a 10-µg/m^3^ increase (Miller et al. 2007).

Although some studies have not found significant differences between men and women, others have reported estimated effects of greater magnitude for women (Brunekreef et al. 2009; Chen et al. 2005; Miller et al. 2007; Zeger et al. 2008). Although the Medicare Cohort Study did not find significant sex differences, the reported mortality risks in the eastern United States associated with a 10-µg/m^3^ increase in PM_2.5_ exposure were slightly higher for women (8.5%; 95% CI, 6.3–10.6%) than men (7.0%; 95% CI, 5.2–8.8%) (Zeger et al. 2008). Risks for cardiovascular outcomes were much higher for the Women’s Health Initiative study, which showed HRs of 2.21 (95% CI, 1.17–4.16) for fatal CHD (definite diagnosis) and 1.24 (95% CI, 1.09–1.41) for total CVD with a 10-µg/m^3^ change in PM_2.5_ (Miller et al. 2007). Average PM_2.5_ exposure (13.5 µg/m^3^) and educational level were lower among this population than for our study of men, but they were comparable overall for many other health risks and behaviors. HRs were also stronger in the Nurses’ Health Study, which used the same exposure estimation methods and geographic area as the current study, although the women generally showed worse health indicators (e.g., less physically active, more current smokers) and lived in areas of lower socioeconomic status (Puett et al. 2008, 2009). The HRs for all-cause mortality were 1.26 (95% CI, 1.02–1.54); 1.03 (95% CI, 0.89–1.18); 1.07 (95% CI, 0.97–1.18) with a 10-µg/m^3^ change in PM_2.5_, PM_10–2.5_, and PM_10_, respectively, and 2.02 (95% CI, 1.07–3.78) for fatal CHD with a 10-µg/m^3^ change in PM_2.5,_ in models adjusting for similar potential confounders (Puett et al. 2009). HRs for nonfatal MI appear to be stronger in the current study of men (Nurses’ Health Study HR = 0.73; 95% CI, 0.48–1.12 for a 10-µg/m^3^ change in PM_2.5_). The Adventist Health Study on the Health Effects of Smog (AHSMOG) cohort study of men and women also reported stronger estimated effects among women for fatal CHD with higher overall levels of PM_10_ and PM_2.5_ exposure than those in the current study (Chen et al. 2005). For a 10-µg/m^3^ change in PM_2.5_, the RR for women was 1.42 (95% CI, 1.06–1.90) and 0.90 (95% CI, 0.76–1.05) for men. RRs associated with PM_10_ and PM_10–2.5_ were also higher than comparable risks for men; however, given the differences in exposure contrasts, results are not directly comparable. However, as for the Nurses and Health Professionals air pollution study comparisons, we cannot rule out the impact of education and lifestyle differences. Men in the AHSMOG study exhibited slightly higher levels of education and physical activity than the women.

Modification of air pollution–associated outcomes by sex is biologically plausible, given that some studies have shown greater fractions of deposition for inhaled particles among women (Bennett et al. 1996; Jaques and Kim 2000; Kim and Hu 1998). As Dockery and Stone (2007) suggest, the evidence may also point to increased susceptibility to air pollution due to an underlying cardiac profile that is more common among women, given that sex differences in the studies described appear more consistent with respect to cardiovascular outcomes. Some studies have shown sex differences in the cardiovascular pathways that air pollution may impact (Hoffmann et al. 2009; Kublickiene and Luksha 2008; Noon et al. 2008; Sztajzel et al. 2008). However, the specific biological mechanisms through which PM air pollution affects all-cause mortality and cerebrovascular and CVD outcomes have not been fully elucidated. Proposed pathways include endothelial dysfunction, oxidative stress, and inflammation (Donaldson et al. 2001; Pope et al. 2004; Pope and Dockery 2006; Utell et al. 2002).

Our GIS-based temporal spatial smoothing models of PM exposures resulted in estimates that fell into the range of exposures reported by previous studies. As previously reported, our models showed high predictive accuracy and precision, with *R*^2^ ranging from 0.62 to 0.77 (Yanosky et al. 2008, 2009). However, the possibility of residual confounding exists, because we did not include such environmental covariates as temperature. Given that the risk sets in the Cox model are updated monthly and we control for state and season in the model, the level of residual confounding is likely to be negligible. We also did not have information on behavioral covariates, such as time spent outside or proportion of time spent at the address, which may lead to misclassification in the personal exposure estimate. Part of the difference between these results and those from the Nurses’ Health Study may be attributable to residential addresses being used for the nurses, whereas a mixture of occupational and residential addresses were available for the health professionals. However, results did not change for the men when the analysis was restricted to known residential addresses.

Findings from the current study may not be generalizable to other populations of men. Our study population represented a narrower range of socioeconomic status and fewer occupational exposures to dust and fumes than the U.S. population as a whole. The Health Professionals Follow-Up Study consisted of health professionals with high levels of educational attainment, physical activity, never smokers, and low-risk diets. Therefore, they are likely to be much healthier than average. The death rate in the general population of U.S. men 65–69 years of age has been estimated to be 2,125 deaths per 100,000 person-years (Centers for Disease Control and Prevention 2007), whereas the rate of nonaccidental deaths in the Health Professionals Follow-up Study is 1,275 per 100,000 person-years. Similarly, the incidence rate of CVD in this cohort (7.8 cases per 1,000 person-years) (National Heart, Lung and Blood Institute 2006) is lower than that reported by the Framingham Heart Study (34.6 cases per 1,000 person-years for men 65–74 years of age), a study more representative of the general population. However, rates could be somewhat more similar than those reported here because of our inability to make exact comparisons with our age strata and time period.

Findings have been somewhat inconsistent regarding the impact of socioeconomic status on relationships between mortality (Bateson and Schwartz 2004; Krewski et al. 2000; Pope et al. 2002), CVD, and air pollution exposures; however, as Bateson and Schwartz (2004) suggest, inconsistencies may be attributable somewhat to differences in the geographic scale of these measures, with individual level measures showing higher risk for lower educational attainment. In the reanalysis of the American Cancer Society Study, men with education above high school showed RRs of all-cause mortality comparable with those found in our study (1.02; 95% CI, 0.89–1.17) (Krewski et al. 2000). Healthier lifestyles may also reduce risks from air pollution, as demonstrated by increased risks of air pollution–associated cardiovascular outcomes among those with larger BMIs (Miller et al. 2007; Puett et al. 2008, 2009). The impact of health behaviors in this population will be examined in detail in further work. In addition, the current study was restricted to the northeastern and midwestern United States, and the addresses used for assigning air pollution exposure estimates were a mixture of work and residential addresses. Differences in findings from the current study compared with some other cohort studies may be partially due to the differences in regional pollutant mixtures, given that other cohort studies covered larger or dissimilar geographic areas of the United States (Dockery et al. 1993; Laden et al. 2006; Pope et al. 2002; Zeger et al. 2008). For example, Zeger et al. (2008) reported stronger risks for the central versus eastern regions of the United States. However, this does not account for the stronger risks found among Nurses’ Health Study cohort participants, as those studies were conducted in the same geographic region as the current study (Puett et al. 2008, 2009).

## Conclusion

Overall, associations of chronic PM exposures with all-cause mortality or CVD outcomes were not evident in this population of male health professionals living in the northeastern and midwestern United States. The lower HRs found in this study should be explored further to ascertain whether men with high socioeconomic status and healthy lifestyles are less susceptible to cardiovascular outcomes associated with long-term particle exposure.
